# Gambling-like Features in fan Tokens

**DOI:** 10.1007/s10899-023-10215-0

**Published:** 2023-05-12

**Authors:** Hibai Lopez-Gonzalez, Mark D. Griffiths

**Affiliations:** 1 Department of Library, Information Science, and Communication, University of Barcelona, Melcior de Palau 140, Barcelona, 08014 Spain; 2 International Gaming Research Unit Psychology Department, Nottingham Trent University, Nottingham, UK

**Keywords:** Fan tokens, gamification, sport, gambling, cryptocurrency, fan exploitation

## Abstract

Fan tokens are a form of cryptocurrency that allow owners to participate in various fan-related experiences such as voting on the music to be played during half-time breaks in sporting events. Since 2020, many elite sport teams have issued fan tokens, allegedly as a way to engage with fans and hear their voice. However, fan tokens also raise some concerns. They are largely gamified digital items that intend to keep fans within the providers’ app. Also, they can be traded in exchange platforms, which arguably transform them into collectibles, whose value can vary over time. Here, we explore fan tokens through a case study from a football (soccer) club (i.e., an F.C. Barcelona fan token). Drawing on literature from situational and structural characteristics of gambling, we analyse the gambling-like features that fan tokens include in their product design. Such features are discussed from a public health perspective, comparing what they mean in gambling contexts and how potentially harmful they could be for fan token holders.

## Introduction

### Overview

Fan tokens in sport are *“a form of cryptocurrency that gives holders access to a variety of fan-related membership perks like voting on club decisions, rewards, merchandise designs and unique experiences”* (Dwyer, 2022, p. 1). Typically, fan tokens are digital assets that allow holders to vote on arguably trivial things such as the music that will be heard during the half-time break of home football games, the design of the corner flags, or a motivational message embroidered on the team captain’s armband. Also, owners can get a small discount in the sports teams’ official store or enter a raffle to meet the team’s star player in person (D’urso, 2021). Fan tokens can also be bought and sold on cryptocurrency exchanges. Purchasing these digital assets does not equate to owning a share of the sport organisation’s capital, nor equate to having rights to attend the games in the stadium.

Fan tokens have been praised as a new source of income for sport organisations, one that complements their traditional streams of revenue (broadcasting rights, matchday revenue, and sponsorship deals; Lopez-Gonzalez et al., 2017), targeting younger audiences and adding an aura of modernity through digitalisation (Kwon & Tae, 2022). To overcome the public scrutiny that might view the issue of fan tokens as a form of extreme mercantilism contrary to the supposedly laudable values of sport, fan token businesses have labelled their products as ‘fan engagement platforms’ that bring fans closer to their teams (Socios, 2022b). Many sports teams issuing their fan tokens in 2022 have reported them selling out within the first few hours of the initial token offering. Usually, these teams release about one million fan tokens for an average price of two euros, resulting in a gross revenue of approximately two million euros in about two hours (Scharnowski et al., 2021). In exchange, sports teams are committed to very low-cost actions for holders, which makes the business of fan tokens very hard to dismiss by sport organisations. Some scholars have argued that the negative economic impact of the COVID-19 pandemic on stadium attendance revenue further accelerated the mass adoption of fan token business as a way of diversifying sports teams’ sources of income (Demir et al., 2022).

Criticism of fan tokens has taken multiple forms. In the UK, the Advertising Standards Authority (ASA) ruled that Arsenal F.C. (Football Club) had to remove their fan token advertisements from social media because they were *“misleading and failed to illustrate the risk of the investment”*, did not make it clear that *“fan tokens are cryptoassets”*, and that they *“took advantage of consumers’ inexperience or credulity”* (Advertising Standards Authority, 2022). Two years earlier, in 2020, Premier League football team West Ham United was forced to cancel the issuing of the club’s official fan tokens for what was perceived by its fans as a *“fan opinion exploitation scheme”* (Football Supporters Association, 2020). Fan tokens are commonly subsumed under the non-fungible tokens (NFTs) umbrella, although they are technically ‘fungible’ because one fan token is identical and interchangeable with another. As such, NFTs and fan tokens have received criticism as tools for the commodification of fandom (Zaucha & Agur, 2022).

Fan tokens have been demonstrated to be highly volatile digital assets, significantly riskier than the most popular cryptocurrencies, and their market valuation tends to be largely dependent on the team’s recent performance (Scharnowski et al., 2021). This means that the fan token market, unlike the traditional stock market, is populated by two distinct personalities with opposite agendas: individuals who see fan tokens as speculative assets, and fans who obtain utility by the mere ownership of tokens, so-called ‘fan-investors’ (Zuber et al., 2005). It is arguable that their cohabitation makes the latter more vulnerable to exploitation by the former, especially in a context sensitive to price manipulation (Chava et al., 2022).

Here, we interrogate fan tokens from a very specific sort of criticism, namely its use of gambling-like features. Fan tokens are not considered gambling products by regulators because they do not adhere to the definition of gambling, which is typically built around three main concepts: (i) an initial stake, (ii) an event with an uncertain outcome, and (iii) a potential reward (Macey & Hamari, 2022). Although fan tokens do not meet these criteria, we argue that they possess attributes that mimic those of gambling products and their commercialisation strategies. This line of argumentation takes elements from the gamblification/gamification literature (Macey & Hamari, 2022; Newall & Weiss-Cohen, 2022) but specifically builds its main arguments on the situational and structural characteristics of gambling. In other words, how environmental factors (*situational*) as well as product design decisions (*structural*) influence the gamblers’ behaviour (e.g., Griffiths 2005; McCormack & Griffiths, 2013).

Utilizing this theoretical framework, we discuss how some fan token features resemble the situational and structural characteristics of gambling, with the implication that utilized as such, they can leave consumers unprotected and cause them harm. The paper is structured using a case study example, namely, the release of fan tokens by the Spanish football club F.C. Barcelona through *Socios.com*. This case study aptly captures the patterns of fan token commercialisation when applied to a world-renowned football club, from the anticipation to its release to the actual characteristics of the token once it is purchased.

### Background on Fan Tokens

Despite the apparent triviality and infancy of fan tokens, their market has become large in volume. *Fan Market Cap* and *Coin Market Cap* – two of the leading trackers of information of fan tokens – estimated the global fan token market capitalisation to be around $380 and $430 million, respectively (Coin Market Cap, 2022; Fan Market Cap, 2022). At present, the great majority of sport organisations in the business of fan tokens are football teams and leagues based in Europe and South America, as well as Formula 1 teams, but these digital assets are rapidly expanding to other sports. A large number of elite football teams in Europe have already released their official fan token collections including Manchester City (UK), Arsenal FC (UK), Paris Saint-Germain (France), Juventus (Italy), A.C. Milan (Italy), Internazionale de Milano (Italy), F.C. Barcelona (Spain), and Atlético de Madrid (Spain), as well as the national football teams from Brazil, Argentina, Spain, Italy, and Portugal.

Fan tokens could be likened to traditional football card collections, and be thought as their digital *tokenization*, although the existence of a secondary (and instant) exchange market in which to speculate with them, and the utility components attached to them, makes fan tokens somewhat different. Others have seen similarities between card collectible environments such as *Magic: The Gathering* and fan tokens (Katwala, 2022), in the way that the two set up a system of collectibles that increase their value over time, with some cards possessing utility value that permits holders to win games, and featuring scarcity mechanisms to control how likely is it for a holder to obtain a specific card.

The mechanics of the issue of fan tokens are simple. A sport organisation announces a deal to release fan tokens in collaboration with a dedicated company. Over the course of a few months, a Fan Token Offering (FTO) will be scheduled. Initially, buyers are only able to purchase a limited number of tokens per person. This phase lasts a few hours. Once it is finished, another phase begins, in which (typically) some previously established buying restrictions no longer apply. Finally, the unsold fan tokens are released with no limit per person. At the initial phase the asking price is fixed, and hitherto it has typically been set at about two euros (approximately two US dollars). Later on, the price depends on supply and demand, as with any other stock value. The issuing company usually caps the maximum number of fan tokens of each team to around 40 million. The acquired tokens can be used for their utility or, conversely, be treated as financial assets and exchanged at the going rate in cryptocurrency exchanges.

The company *Socios.com* is credited to be the introducer of the business concept of fan tokens. So far, *Socios.com* (and to a lesser extent, *Binance*) have been the two companies responsible for the vast majority of the fan tokens in the market. *Socios.com* works in cooperation with *Chiliz* – a blockchain financial technology firm – and both are subsidiaries of *Mediarex Group* (Socios, 2022b). Chiliz procures the technological foundation for *Socios.com*, and issues the cryptocurrency chiliz ($CHZ), without which fan tokens cannot be bought. The *Socios.com* brand has become ubiquitous in world sport in under two years. In 2021, the football clubs Valencia C.F. (Spain) and Internazionale de Milano (Italy) showed the logo of *Socios.com* on their jerseys. As of 2022, *Socios.com* has sealed commercial deals with multiple National Basketball Association (NBA), National Football League (NFL), Major League Soccer (MLS) franchises in the USA, 64 European and Latin American football teams, as well as other organisations in tennis, motorsports, and fighting sports. *Socios.com* sponsors Ballon d’Or football awards and have also signed Argentinian footballer Lionel Messi as the company’s brand ambassador. F.C. Barcelona reported that *Chiliz* had acquired 25% of their digital studio to produce NFTs and metaverse projects for the team (Metzger, 2022).

Fan tokens along with NFTs are a group of cryptoassets that run blockchain-based digital products. These cryptoassets can be traded in cryptocurrency exchange platforms, just like any other investment product. As an indicator of their ubiquity, a study analysing the sponsorship of English Premier League football teams identified an emerging trend of financial trading apps and cryptocurrencies visible on the teams’ jerseys (Newall & Xiao., 2021). A simple *Google* search with keywords such as crypto, sport, sponsor, and trading provides a clear picture of the abundance of professional athletes engaged in the sponsorship of crypto and trading brands. These include (sponsored brand in parenthesis): Cristiano Ronaldo (*XTrade* and *Binance*), Gaël Monfils, Joe Cole, Elina Svitolina (*eToro*), Ronaldinho (*Olymp Trade*), Venus and Serena Williams (*Shares*), Conor McGregor (*Tiger Trade*), Gianluigi Buffon (*TMGM*), Usain Bolt (*Ava Trade*), Kylian Mbappé (*Sorare*), Stephen Curry, Shaquille O’Neal, Naomi Osaka, and Tom Brady (*FTX*), Kevin De Bruyne (*Phemex*), Daniel Ricciardo (*EightCap* and *OKX*), Pep Guardiola (*CFI Dubai*), Victor Moses (*Binomo*), Steve Smith (*Trade360*), Ronaldo Nazário (*Betfair*), Andrés Iniesta (*UFX*), LeBron James (*Crypto.com*), Kevin Durant (*Coinbase*), Giannis Antetokounmpo and Neymar Jr. (*NFTSTAR*), and Michael Jordan (*Heir*).

### The Situational and Structural Characteristics of Gambling

Gambling-like features of fan tokens are explored in the present paper through the lens of a gambling theory that categorises in three groups the factors that affect individuals to gamble: individual, situational, and structural factors (Griffiths, 2005). Individual factors involve determinants of personal vulnerability, including personality traits, financial motivations, and biological characteristics, and personal beliefs and attitudes. However, the role of individual factors is a point of dispute. When researchers focus on individual factors, they often try to explain gambling behaviour as a fundamentally individualized behaviour in which the gambler needs to be responsible for their actions. Some scholars contend that individual factors models focus too much on the responsibility of the gambler to avoid excessive gambling, disproportionately placing the blame on the gamblers. These scholars contend that gambling providers should be held accountable for releasing harmful products (Hancock & Smith, 2017; Miller & Thomas, 2018). Conversely, other scholars believe this is a mischaracterisation of the individuals factors model, and that the personal responsibility of gamblers should not be ignored (Shaffer & Ladouceur, 2021). In any case, for the present study, individual factors will not be considered as the paper focuses on the actions carried out by the fan token industry, and not on the behaviour of fan token holders.

In this paper we focus upon situational and structural characteristics – also called ‘object exposure’ and ‘object interaction’, respectively, by other authors (Shaffer et al., 2004) – because they are solely or partly attributable to the gambling industry, and by juxtaposition in the present paper, to fan token providers. Situational characteristics refer to factors present in the environment that facilitate gambling involvement (e.g., the legal framework for gambling in a given jurisdiction, how available the product is for consumers, and the advertising and marketing of the product). Structural characteristics, in turn, refer to factors that have to do with the design of the gambling products (e.g., reward structure, duration, cost of playing, speed, and frequency of play), which try to maximise the gamblers’ time on the activity (Dow Schüll, 2012). As aforementioned (and unlike individual factors), situational and structural factors can be traced back to the gambling providers, and therefore build a case for the co-responsibility of the gambling industry in the acquisition, development, and maintenance of gambling-related harms (Binde, 2007; Yani-de-Soriano et al., 2012).

## Method

### Procedure

We downloaded the *Socios.com* app, opened an account and exchanged some euros (real currency) for chiliz (cryptocurrency) in order to buy a fan token. Only one fan token from one team was purchased for two main reasons. First, all fan token releases appear to be almost identical in terms of the phases and number of tokens made available in each phase, the initial price of the tokens, and the advertised *utility* that will bring the purchase of the token. Second, owning one fan token gives the same access within the *Socios.com* app as owning more than one fan token. That is, although holding multiple tokens gives access to special rewards, greater discounts, and a heavier weight in pools, such promotions and rewards are visible for owners of a single token. Among the top European football teams eligible, an F.C. Barcelona football team fan token was bought for personal preference. We paid a market price of 27.53 chiliz ($CHZ) (≈ €6.05 at the time of writing), which included a small commission fee.

For a period of one month in October 2022, we scanned the *Android* app and its website version in order to identify features that met the parameters of the present study. The app included an augmented reality feature that allows users to ‘hunt’ for rewards in real-world locations, similar to the feature popularised by *Pokémon Go* a few years ago. Additionally, when signing up in *Socios.com*, we accepted to receive commercial communications via email, which were used to complement the analysis. Also, we sent an inquiry to the *Socios.com* customer service platform to ask for the whitepaper of F.C. Barcelona, which is the document that contains the conditions and business model of the token offered. Although the inquiry was responded to, the whitepaper itself had not been received at the time of writing. Alternatively, the original promotional articles released by *Socios.com* in 2020 to promote F.C. Barcelona’s fan tokens were collected by using the *medium.com* search engine, which keeps a copy of the original articles (Medium, 2022). These materials included press articles as well as a 57-second promotional video composed of stock images shared on social media by *Socios.com* and F.C. Barcelona. Finally, we joined the *Chiliz/Socios* official *Telegram* account for Spain to gain access to the conversation that fan token holders were having there.

As a means of comparison, once the analysis was concluded, we bought two additional fan tokens to contextualize whether the F.C. Barcelona token findings were only applicable to that specific token or generalizable to the product category. To that end, we bought one São Paolo F.C. token ($CHZ 1.91) and one Aston Martin Cognizant Formula 1 token ($CHZ 2.71). The functionalities (e.g., quizzes, pools, rewards, et cetera) for São Paolo F.C. token were identical to those of F.C. Barcelona token. For Aston Martin Cognizant, leaderboards were not displayed but the rest of the features were also identical. The Spanish Telegram group did not discuss these two new tokens. Therefore, we concluded that the F.C. Barcelona fan token was an apt illustration of how fan tokens worked at the time of the study.

### Analytical Framework

To systematically explore the gambling-like features in fan tokens, a list of situational and structural characteristics was compiled consulting several sources (i.e., Griffiths 1993; McCormack & Griffiths, 2013; Parke & Griffiths, 2007; Shaffer et al., 2004). The terminology in those lists is sometimes imprecise, and many characteristics overlap or partially overlap with other authors’ classifications. The longest classification, offered by McCormack and Griffiths (2013), lists 64 very concrete situational and structural characteristics but (i) many cannot be applied to fan tokens, (ii) some can be easily condensed under broader categories, and (iii) many are not exclusive of gambling or gambling-like products and can be found in numerous categories of products (e.g., use of colour, naming, online customer tracking, multi-lingual sites, accessibility, availability, etc.). Although these characteristics could be used to promote the use of gambling products and fan tokens, they cannot be construed as being gambling-like features per se, nor do they constitute a case of a *gamblified* experience. Finally, we ended up with a final list of three situational characteristics (regulation and legitimacy issues, marketing and advertising strategies, and social facilitation), and five structural characteristics (intermittent rewards, exacerbation of the time on device, gambling, size of stake, and psychological value of money) that we detail below.

#### Regulation and Legitimacy Issues

Many jurisdictions introduced legislation in the first two decades of the 21st century to adapt laws conceived when venue-based (offline) gambling was the only choice before the advent of internet gambling. During the crafting of these regulations, the gambling industry lobbied for balance between two demands. On one hand, legislators wanted to regulate gambling in such a way that companies would have to apply for licenses. Attached to these licenses would be (and currently are) a number of requirements that would, among other benefits, protect consumers from any gambling company’s wrongdoings (e.g., permitting access to a gambling venue to a self-excluded person). Such license requirements will impose limits to applicants but also differentiate them from illegal gambling providers operating from offshore territories. On the other hand, it was important to design regulations such that the legal requirements were not too restrictive, and that legal gambling options did not become less attractive to customers than illegal (i.e., unlicensed) ones (Orford, 2020).

#### Marketing and Advertising Strategies

The persuasive communication of fan tokens is intimately related to its legitimacy as a legal and socially desirable consumer product. As a relatively new product, advertising communication is crucial to inform public debate about how fan tokens should be perceived according to its providers.

#### Social Facilitation

In gambling contexts, social facilitation can come in the form of peer pressure by friends (Deans et al., 2017), parents (Gay et al., 2016), workmates (Lopez-Gonzalez et al., 2021) or other individuals gambling in the same venue (Rockloff & Dyer, 2007). If the engagement in a given activity is wide enough in a society, social facilitation occurs naturally but companies can accentuate it by means of, for instance, influencer marketing or sponsorships. Social facilitation is a situational characteristic that affects consumer behaviour but occurs over multiple product types and is not idiosyncratic of gambling. However, a few social facilitation techniques are specific to gambling, and we have seen some of these in fan token products.

#### Intermittent Rewards

Gambling is profitable because, among other reasons, it leverages animal learning principles to design products that are difficult to stop using. Consequently, some of the most studied principles in gambling research deal with how gambling rewards affect gamblers’ behaviour (Ramnerö et al., 2019; Skinner, 1971). Gambling products typically feature intermittent reward mechanisms, which reinforce behaviour in a randomised manner or following other logics that maximise the gamblers’ engagement with the product (Horsley et al., 2012). These are well-established mechanisms that produce a steady financial income for the gambling industry.

#### Exacerbation of the Time on Device

Gambling industry insiders popularised the term ‘time on device’ (TOD) as the ultimate goal of slot machines designers (i.e., the more the user stays on device, the higher the revenue for the provider; Dow Schüll 2012). For gambling operators, the TOD can work as a reliable proxy for gambling revenue because gambling products procure on average long-term losses for gamblers based on a negative expected return (Rachlin et al., 2015). The concept of TOD has gained wider traction and is now being applied to streaming platforms such as *Netflix* (Sinclair & Clark, 2021) and virtually any digital design that aims to capture the attention of consumers (Bartlett, 2019).

#### Gambling

In this context, gambling refers to the use of gambling activities in non-gambling environments such as the insertion of casinos in videogames targeting adolescents (King et al., 2014).

#### Size of Stake

The debate about the comparative risks involved in gambling with higher versus lower stake sizes remains inconclusive. Higher stakes could mean more severe gambling problems, but lower stakes could also mean higher participation, which will increase the number of total gamblers, especially those of younger age (e.g., Parke 2009).

From a public health perspective, a small stake size in a product considered attractive for young consumers could be dangerous if such product could lead to the consumption of another, more harmful product. This is known as the ‘gateway drug’ hypothesis. In the field of gambling, the gateway hypothesis has been studied regarding the association between videogame playing and gambling (Delfabbro & King, 2020), and loot boxes and gambling (Zendle & Cairns, 2018).

#### Psychological Value of Money

The gambling research field has long considered how different representations of money can affect gambling behaviour. Poker chips, as substitutes of real money in poker, could help to lower the psychological value of money and minimise the ’pain of paying’ (Lapuz & Griffiths, 2010). In general, it is accepted that money formats have an effect on gamblers’ perception of the psychological value of money, and that format manipulation by gambling providers can increase gambling expenditure (see e.g., Limbrick-Oldfield et al., 2022; Palmer et al., 2022). Scholars studying the purchase of virtual items on videogames have started to pay attention to the effects of numerosity. Numerosity is defined as ‘the number of units into which a stimulus is divided’ (Pelham et al., 1994). They argue that the use of ‘uncommonly divisible quantities prevent players from making intuitive value estimations’ (Scholten et al., 2019, p. 381). It is yet to be understood how exactly numerosity effects work but it appears clear that videogame publishers are trying to leverage in-game currency formats, scarcity, and numerosity to increase purchases (Huang et al., 2020). A similar strategy could be argued to be in place for fan tokens.

## Results and Discussion

### Gambling-like Situational Characteristics in Fan Tokens

#### Regulation and Legitimacy Issues

The current legal situation of fan tokens echoes specific aspects observed in the gambling regulation context. First, as in unregulated gambling markets, fan tokens’ markets are subject to price manipulation, with investors heavily promoting their ownership of tokens on social media just to make the price rise and sell for a profit (Davies, 2022). Second, fan tokens are not guaranteed by traditional financial systems and governments and the discontinuation (for bankruptcy or other reasons) of the company releasing the tokens might render the tokens useless. This was the situation with illegal gambling firms that could not be held accountable by any central authority to honouring their payments agreements to customers with successful bets. Third, fan token products are not subject to responsibility messages, disclaimers, and warnings, although they introduce an asset that can be traded for money and its value is dependent on fluctuations on the market, which means the same requirements as for financial products should apply, as exemplified by the ruling of ASA in the case of Arsenal F.C. fan tokens (Advertising Standards Authority, 2022). Other examples can be found in the recent Manchester City partnership with finance trading company *3Key*, a company with no digital footprint that forced the team to cancel the deal within days to curb the backlash it created (MacInnes, 2022). Four, the corporate discourse about fan tokens is starting to resemble that of the risks of gambling products. For instance, a spokesperson for Arsenal F.C. told reporters: *“we advise fans not to spend more on fan tokens than they can afford”* (D’urso, 2021). This way of talking is similar to what the gambling industry has been using for decades, and appears to translate into fan token terminology the first question of the popular Problem Gambling Severity Index “*Have you bet more than you could really afford to lose?*”, a screening instrument for gambling problems (Ferris & Wynne, 2001).

#### Marketing and Advertising Strategies

The current analysis has shown a number of similarities between the advertising and marketing strategies of gambling and fan tokens. First, fan tokens struggle to clearly communicate whether they are a product designed for entertainment or for making money. In an interview with Alexandre Dreyfus, CEO of *Socios.com*, he emphasised that fan tokens *“are for entertainment, not investment”* and that trading *“is not their primary function*” (McCaskill, 2022). However, some communicative actions of *Socios.com* somewhat contradict this. In 2021, *Socios.com* promised to ‘burn’ 20,000 F.C. Barcelona tokens for every goal the team scored, and burn 40,000 for each win. In their promotional message, *Socios.com* stated: “*Burning is a very common practice in crypto, and is simply a way to reduce the circulating supply of a token meaning the tokens become more scarce*” (Socios, 2021). The implication of an asset becoming scarcer is that it will gain market value. This practice does not fit well with the alleged nature of fan tokens, as a motivation purely based on entertainment would be unaffected by the total supply of fan tokens. Moreover, token value can also be dependent upon the functionalities that the issuer conveys to the product. Initial functionalities when the token was purchased (e.g., types of quizzes, rewards, binding pools) might be substantially modified in the future by subtracting or adding features, therefore altering the underlying value of the asset in a way that fans could not have anticipated. Consequently, not only the market value but also the utility value of the fan token can fluctuate over time, because unlike traditional products who have a defined final shape at the time of the purchase, fan token can vary in their characteristics and functionalities.

The entertainment versus investment product categorisation is also a classic dilemma in gambling communication, and an essential one in order to remove the negative connotations traditionally associated with vice, crime, and the social stigma of experiencing gambling problems (Lang & Rosenberg, 2017). Gambling operators have sought to eradicate from the public imagery the perception of gambling as a demerit good, that is, something *“intrinsically unhealthy, degrading or socially damaging”* (McCormick & Stone, 2007, p. 162). Fan token providers seem to have realised that other cryptoassets such as cryptocurrencies or NFTs face greater reputational risks, mainly because they cannot be construed as anything other than highly speculative assets. Unsurprisingly, in an effort to distance their product from such negatively connoted cryptoassets, *Socios.com* and *Binance* have made it clear that fan tokens are *utility* tokens because they give access to digital services that go beyond the sole ownership of the token (i.e., only for speculative purposes). All things considered, at present there are limited data to discern whether fan tokens are primarily bought and utilised by fans to obtain entertainment or, alternatively, by investors to obtain profit.

Second, fan tokens and other cryptoassets are colonising marketing spaces previously dominated by sports betting advertising. Newall and Xiao (2021) highlighted the fact that the emergence of crypto-gambling and trading sponsorship coincides in time with the implementation of stricter regulation of gambling sponsorships throughout European territories. We contend that the substitution of gambling brands for cryptoassets points in the direction of a similarity of product category, with companies involved in the substitution aiming at a similar target audience in terms of demographics and interests. If this assumption is correct, then fan tokens will become symbolically subsumed under the same category as cryptocurrencies and trading platforms, and their efforts to present to the public their product as something different (and more benign) will likely be unfruitful.

Finally, fan token communication delivers the message that buying their product equals to being (or leads to becoming) a better F.C. Barcelona fan. This is an instance of the use of the conceptual metaphor of love already that has already been empirically shown in sports betting advertising (Lopez-Gonzalez et al., 2018). The Conceptual Metaphor Theory explains metaphors as *“understanding and experiencing one kind of thing in terms of another”* (Lakoff & Johnson, 1980, p. 5). By drawing from the metaphorical realm of love, fan token promotions communicate the idea that buying tokens is an act of love towards the team. Analysed communications repeatedly show the expression *“getting closer to the team”* as something that can be achieved by buying fan tokens. Conversely, following the metaphor, not buying them would be an act of separation or of not showing true love towards the team.

#### Social Facilitation

In the analysis, we noted the use of ‘leaderboards’. A leaderboard ranks users for other users to see. Holders of fan tokens are encouraged to participate in pools, result predictions, quizzes, and spending time playing in their in-app games in order to earn points for the leaderboard. Leaderboards are prominently displayed on the *Socios.com* app and they have a significant relevance for users in two ways. Firstly, token holders compete to gain access to special offers and rewards by outscoring other token holders. Secondly, a high leaderboard position implies social recognition by the peer group and a good fan status. These two observations are based on the analysis of the *Telegram* group, in which leaderboards appear to constitute the central topic of discussion.

Leaderboards also allow holders to brag about their ranking. In one of their messages, *Socios.com* said: “*Imagine being able to show off to your friends and fellow Barça supporters that you are the number one Barça Fan in your country or city!*”. This resembles the potentially exploitative practices of gambling operators at least in two different ways. First, bookmakers have been known to display ‘lists of winners’ inside betting shops, ranking those winners by the amount of money they have won. A qualitative research study found this method to annoy gamblers experiencing problems (Lopez-Gonzalez et al., 2020). Second, leaderboards can be promoted in a way that spending more time on a platform equals to being a better fan of a team. In fact, *Socios.com* claims in its website *“Everyone brags about being their team’s biggest fan, now it’s time to prove it! Compete in our leaderboards and get ready to claim epic rewards”.* Such a way of framing the meaning of engagement resembles that of sports betting operators, in that it presents the use of their proprietary app as a direct reflection of true fandom and loyalty to a team (Deans et al., 2016; Lopez-Gonzalez & Griffiths, 2016). Additionally, the claim by *Socios.com* adds to the idea of leaderboards as ‘bragging places’, which connects to research concerning lifestyle consumption communities of sports betting made in Australia in which engaging more in sports betting was found to be associated with bettors having greater bragging rights towards their peers (Gordon et al., 2015).

### Gambling-like Structural Characteristics in Fan Tokens

#### Intermittent Rewards

The app of *Socios.com* is designed in a way that builds a complex reward system for fan token holders. Rewards are structured on different levels within *Socios.com* app. High value rewards such as meeting a professional player or winning tickets to a sports game are scarce, and consequently intermittent, and they often require holding a minimum number of tokens. Such rewards are not randomised but sequenced in a way that individuals holding more fan tokens and engaging more on the platform have a higher chance of winning them. The gamified experience also includes other lower value rewards such as discounts on merchandising and being able to participate in binding pools (i.e., pools whose results must be respected by the team) which are fixed rewards. From the analyses performed on the app and other documentation the reward volatility could not be estimated (i.e., more volatility means less frequent but higher value wins; Parke & Parke 2013).

Max Rabinovitch, Chief Strategy Officer for *Socios.com* and *Chiliz* says in one of the documents: “*Creating a deeper, more gamified team by team experience for fans in our platform is integral to our vision of creating an engagement and rewards ecosystem for Fan Token holders that truly hits a critical mass in terms of daily value and reasons to participate*” (Socios, 2022a). It is telling the acknowledgement that rewards are not individual items but parts of a greater system – an ecosystem – that fuels participation. An attractive reward ecosystem is essential to *Socios.com* because it keeps fan token holders engaged in the platform while it distances its product from other merely speculative cryptoassets, solidifying its perception as a utility token.

#### The Exacerbation of the Time on Device

*Socios.com* features multiple strategies to maximise the time fan token holders spend on its app. The maximization of TOD on *Socios.com* is built through an intricate network of activities and rewards. Fan token holders get their TOD rewarded by: (i) doing check-ins when a match is played, which is a way of communicating to the app that you are watching the game, (ii) participating in the free-kick challenge, which is a mini-game available for those who checked-in in the first place, to be played during half-time breaks in matches, (iii) simply by logging in the app at least once every 24 h, (iv) voting on pools, (v) predicting the score in a match, (vi) redeeming vouchers, collectibles, or any other fan reward, (vii) participating in games and quizzes, (viii) hunting tokens in the augmented reality feature, and (ix) buying or selling fan tokens. These activities obtain currency rewards, which can come in the form of (a) points, (b) experience points (XP), (c) free fan tokens, and (d) SSU Loyalty Tokens, which is a native *Socios.com* cryptocurrency with no exchange value. Such currency rewards can be redeemed for other real-world rewards such as meeting players, merchandising, discounts, tickets, etc. Holders can get milestones, trophies, and achievements, in the platform’s terminology. The multi-layered ranking of users creates VIP fan token holders at the top of the scale (i.e., leaderboard), accompanied by some prerogatives but also subject to greater pressure to stay on the platform and to not cease engaging. VIP schemes in gambling contexts have attracted some attention, and since 2020 are regulated in some jurisdictions (such as the UK) to minimise consumer exploitation (Davies, 2020; Gambling Commission, 2020).

As a gamified experience, the environment of *Socios.com* is designed to maximize the TOD while rewarding (psychologically or otherwise) the users for it. The group chat in Spanish on *Telegram* reflects the anxiety some fan token holders feel about the tactics used to engage them. Some complain that they cannot disconnect for a day without being penalised and share links to vote on pools or predict scores so that they do not miss out on these opportunities and descend on the leaderboard.

A previous investigation of the *Socios/Chiliz Telegram* group in English showed that the conversations there had no relationship with sport and that they all revolved around financial issues (D’urso, 2021). In the present study, the Spanish version *Telegram* group showed similar results, with the focus being on sharing ways of earning more points to climb the ladders proposed by the platform. Interestingly, some fan token holders seemed to play the role of ‘tipsters’ in sports betting contexts, recommending others to buy more tokens now. Given the speculative nature of the asset, current fan token holders are incentivised to promote the product they bought in order to increase its demand, and consequently its price. This is very similar to what other strategies employed by cryptocurrencies (and catalogued as Ponzi schemes) have been doing (Segal, 2022).

#### Gambling

The most obvious gambling-like feature within *Socios.com* app is a sport predictor functionality similar to a sports betting website. Fan token holders are encouraged to predict the winners, scorers, and the final result of matches. However, holders who engage with the predictor do not stake any money or other type of asset, and cannot win money in return. The reward for users is that correct predictions get awarded points, which in turn promote users within leaderboards and make them more likely to win other rewards.

#### Size of Stake

The fan token business is conceived in way that arguably manipulates the perception of how costly it is to enter it. In the initial coin offerings, the cost of becoming a fan token holder is approximately two euros. This is a very affordable price for all age groups. The theory of fan token consumption as a ‘gateway drug’ could make sense if highly volatile and speculative investments are considered ‘the final drug’, something that aligns well with the ASA’s characterisation of fan tokens as investment products (Advertising Standards Authority, 2022). Fan tokens are listed on exchange platforms and their value fluctuates in reaction to new information and the behaviour of other fan token owners. To the best of our knowledge, no research has established an association between fan token ownership and participation in high-risk investments, but if such relationship existed, fan tokens would have to be viewed as a potentially more problematic consumer product.

#### Psychological Value of Money

The fact that fan tokens can only be bought in $CHZ, the cryptocurrency of *Chiliz* company, also has implications for consumer protection. The F.C. Barcelona fan token we used in the present study was purchased on October 7, 2022, for 26.3$CHZ, plus a commission of 1.23$CHZ. This purchase implies at least a three-degree conversion of money: from digitally stored euros in a bank account to cryptocurrency $CHZ, and from $CHZ to a fan token with its own price fluctuations and market valuation.

Furthermore, the use of $CHZ to buy fan tokens brings an additional concern. The Euro conversion rate at the moment of the fan token purchase documented on the present paper was €0.22 = 1 $CHZ. The conversion of $CHZ to popular Western fiat currencies such as British pounds, US dollars, and Euros is hard to mentally calculate, and obfuscates the real cost of purchasing fan tokens. In simple terms, a user can get more than four $CHZ for a euro. This calls for a numerosity effect. Four $CHZ is four times more units than one euro. The use of $CHZ could implicate a reduced perception of cost (or equivalently, an overestimation of the value of $CHZ).

## Conclusions

In this paper we explored the inclusion of gambling-life features in fan token products. Using as a case study the fan tokens released by F.C. Barcelona and *Socios.com*, the analysis showed that the product design includes a number of characteristics similar to (or borrowed from) gambling products. As a product that explicitly intends to be gamified, fan tokens take pride in including features that increase user participation and minimise the time away from the platform. However, we outlined reasons why such gamification could be potentially harmful.

Fan tokens are digital products allegedly designed to foster fan engagement and they are promoted and released in conjunction with big sport properties. The promotional materials and app features indicate that fan tokens are being presented in a way that fans might think that buying them is a prerequisite for being a good fan. The conversion of fan sentiment into a purchasing decision could be construed as fan exploitation, with the implicit assumption that fans not buying tokens are somehow lesser fans. For sports organisations, issuing fan tokens might become a liability if tokens alienate fans rather than engaging them in meaningful manners.

As cryptoassets with exchange value, fan tokens cannot escape the characterisation of investment products. Possibly because of their relative newness, fan tokens do not seem to suffer from the same mixed public perception as NFTs or cryptocurrencies, but this could rapidly change. Currently, the regulations that protect consumers when considering investment products do not apply to fan tokens, and providers seem to be keen to unequivocally introduce fan tokens as entertainment rather than investment. Regardless of their agenda, fan token holders will dictate what the product really is and how it is consumed, and early indications from chat groups, ASA rulings, and the extant (mainly non-academic) literature suggest that fan tokens will have to be considered trading products in the near future. If that occurs, regulation will have to contemplate how the gamification/gamblification of fan tokens exposes consumers to harms, making them dependent on the rewards of their platforms and subject to the volatility of the asset, risking losing their money. Also, it will have to determine whether fan tokens predominantly allure adolescent and young consumers, who merit special measures of protection.

## Data Availability

No supplementary data is available for this study.

## References

[CR1] Advertising Standards Authority (2022). *ASA ruling on Arsenal Football Club plc*. Retrieved April 29, 2023: https://www.asa.org.uk/rulings/arsenal-football-club-plc-a21-1121873-arsenal-football-club-plc-1.html

[CR2] Bartlett V (2019). Digital design and time on device; how aesthetic experience can help to illuminate the psychological impact of living in a digital culture. Digital Creativity.

[CR3] Binde P (2007). Selling dreams—causing nightmares?. Journal of Gambling Issues.

[CR4] Chava, S., Hu, F., & Paradkar, N. (2022). Gambling on crypto tokens? *Georgia Tech Scheller College of Business*, *SSRN* (Preprint), 4149937. 10.2139/ssrn.4149937

[CR5] Coin Market Cap (2022). Top fan token by market capitalization. Retrieved April 29, 2023, from: https://coinmarketcap.com/view/fan-token/

[CR6] D’urso, J. (2021). Special investigation: Socios ‘fan tokens’ – what they really are and how they work. *The Athletic* Retrieved April 29, 2023, from:https://theathletic.com/2774492/2021/08/18/investigation-socios-fan-tokens-what-they-really-are-and-how-they-work/

[CR7] Davies, R. (2020). ‘It keeps you coming back’: The rise of VIP gambling schemes. *The Guardian* Retrieved April 29, 2023, from:https://www.theguardian.com/society/2020/jan/02/it-keeps-you-coming-back-the-rise-of-vip-gambling-schemes

[CR8] Davies, R. (2022). Dangerous game? Football clubs look to mine fans’ cash with crypto offerings. *The Guardian*. Retrieved April 29, 2023, from: https://www.theguardian.com/technology/2022/jan/22/dangerous-game-football-clubs-look-to-mine-fans-cash-with-crypto-offerings

[CR10] Deans EG, Thomas SL, Daube M, Derevensky J, Gordon R (2016). Creating symbolic cultures of consumption: An analysis of the content of sports wagering advertisements in Australia. Bmc Public Health.

[CR9] Deans EG, Thomas SL, Daube M, Derevensky J (2017). The role of peer influences on the normalisation of sports wagering: A qualitative study of australian men. Addiction Research & Theory.

[CR11] Delfabbro P, King DL (2020). Gaming-gambling convergence: Evaluating evidence for the ‘gateway’ hypothesis. International Gambling Studies.

[CR12] Demir E, Ersan O, Popesko B (2022). Are fan tokens fan tokens?. Finance Research Letters.

[CR13] Dow Schüll, N. (2012). *Addiction by design: Machine gambling in Las Vegas*. Princeton University Press. 10.2307/j.ctt12f4d0.

[CR14] Dwyer, K. (2022). *What are fan tokens? CoinMarketCap* Retrieved April 29, 2023, from: https://coinmarketcap.com/alexandria/article/what-are-fan-tokens

[CR15] Fan Market Cap (2022). *Fan market cap - Fan tokens*. Retrieved April 29, 2023, from: https://www.fanmarketcap.com/

[CR16] Ferris, J., & Wynne, H. (2001). *The canadian Problem Gambling Index*. Canadian Centre on Substance Abuse.

[CR17] Football Supporters Association (2020). *West Ham end controversial blockchain partnership*. Retrieved April 29, 2023, from: https://thefsa.org.uk/news/west-ham-end-controversial-blockchain-partnership/

[CR18] Gambling Commission (2020). *High value customers: Industry guidance*. Gambling Commission. Retrieved April 29, 2023, from: https://assets.ctfassets.net/j16ev64qyf6l/4TVcLLR9ymZoEKr5fC4JCQ/3c22f256c91eb8452c35a37ca723d5f6/Guidance_to_operators_on_high_value_customers.pdf

[CR19] Gay, J., Gill, P. R., & Corboy, D. (2016). Parental and peer influences on emerging adult problem gambling: Does exposure to problem gambling reduce stigmatizing perceptions and increase vulnerability? *Journal of Gambling Issues*, 30. 10.4309/jgi.2016.33.3. *33*.

[CR20] Gordon R, Gurrieri L, Chapman M (2015). Broadening an understanding of problem gambling: The lifestyle consumption community of sports betting. Journal of Business Research.

[CR21] Griffiths MD (1993). Fruit machine gambling: The importance of structural characteristics. Journal of Gambling Studies.

[CR22] Griffiths MD (2005). A biopsychosocial approach to addiction. Psyke & Logos.

[CR23] Hancock L, Smith G (2017). Critiquing the Reno model I-IV international influence on regulators and governments (2004–2015) — the distorted reality of “responsible gambling. International Journal of Mental Health and Addiction.

[CR24] Horsley RR, Osborne M, Norman C, Wells T (2012). High-frequency gamblers show increased resistance to extinction following partial reinforcement. Behavioural Brain Research.

[CR25] Huang Y, Lim KH, Lin Z (2020). Leveraging the numerosity effect to influence perceived expensiveness of virtual items. Information Systems Research.

[CR26] Katwala, A. (2022). NFTs are conquering soccer. *Wired*. Retrieved April 29, 2023, from: https://www.wired.co.uk/article/nfts-conquering-soccer

[CR27] King DL, Delfabbro PH, Kaptsis D, Zwaans T (2014). Adolescent simulated gambling via digital and social media: An emerging problem. Computers in Human Behavior.

[CR28] Kwon, J. H., & Tae, J. H. (2022). NFT contents enjoyment and policy suggestions - focusing on digital aura concept. *Journal of the Korea Entertainment Industry Association*, *16*(5), 10.21184/jkeia.2022.7.16.5.155.

[CR29] Lakoff, G., & Johnson, M. (1980). *Metaphors we live by*. The University of Chicago Press.

[CR30] Lang B, Rosenberg H (2017). Public perceptions of behavioral and substance addictions. Psychology of Addictive Behaviors.

[CR31] Lapuz J, Griffiths M (2010). The role of chips in poker gambling: An empirical pilot study. Gambling Research.

[CR32] Limbrick-Oldfield EH, Chua C, Cringle N, MacDonald K, Ferrari MA, Zhang K, Clark L (2022). Cashless gambling and the pain of paying: Effects of monetary format on slot machine gambling. Addiction Research & Theory.

[CR33] Lopez-Gonzalez H, Griffiths MD (2016). Understanding the convergence of online sports betting markets. International Review for the Sociology of Sport.

[CR37] Lopez-Gonzalez, H., Stavros, C., & Smith, A. C. T. (2017). Broadcasting sport: Analogue markets and digital rights. *International Communication Gazette*, *79*(2), 10.1177/1748048517694969.

[CR35] Lopez-Gonzalez H, Guerrero-Solé F, Estévez A, Griffiths MD (2018). Betting is loving and bettors are predators: A conceptual metaphor approach to online sports betting advertising. Journal of Gambling Studies.

[CR34] Lopez-Gonzalez H, Griffiths MDMD, Jimenez-Murcia S, Estevez A, Estévez A (2020). The perceived influence of sports betting marketing techniques on disordered gamblers in treatment. European Sport Management Quarterly.

[CR36] Lopez-Gonzalez H, Jiménez-Murcia S, Griffiths MD (2021). The erosion of nongambling spheres by smartphone gambling: A qualitative study on workplace and domestic disordered gambling. Mobile Media & Communication.

[CR38] Macey, J., & Hamari, J. (2022). Gamblification: A definition. *New Media & Society*, 14614448221083904. 10.1177/14614448221083903.

[CR39] MacInnes, P. (2022). Manchester City sign deal with mysterious cryptocurrency start-up. *The Guardian* Retrieved April 29, 2023, from: https://www.theguardian.com/football/2021/nov/17/manchester-city-sign-partnership-deal-with-cryptocurrency-start-up

[CR40] McCaskill, S. (2022). Socios CEO: Fan tokens are for entertainment, not investment. *Sports Promedia* Retrieved April 29, 2023, from: https://www.sportspromedia.com/news/socios-ceo-alexandre-dreyfus-fan-tokens/

[CR41] McCormack A, Griffiths MD (2013). A scoping study of the structural and situational characteristics of internet gambling. International Journal of Cyber Behavior Psychology and Learning.

[CR42] McCormick B, Stone I (2007). Economic costs of obesity and the case for government intervention. Obesity Reviews.

[CR43] Medium. (2022). *Medium*. Medium.Com.

[CR44] Metzger, A. (2022). *Crypto firm Chiliz nabs $100 M stake in FC Barcelona’s digital studio*. *Bit Courier* Retrieved April 29, 2023, from:https://bitcourier.co.uk/news/chiliz-aug2022

[CR45] Miller HE, Thomas SL (2018). The problem with ‘responsible gambling’: Impact of government and industry discourses on feelings of felt and enacted stigma in people who experience problems with gambling. Addiction Research and Theory.

[CR46] Newall PWS, Weiss-Cohen L (2022). The gamblification of investing: How a new generation of investors is being born to lose. International Journal of Environmental Research and Public Health.

[CR47] Newall PWS, Xiao LY (2021). Gambling marketing bans in professional sports neglect the risks posed by financial trading apps and cryptocurrencies. Gaming Law Review.

[CR48] Orford, J. (2020). *The gambling establishment: Challenging the power of the modern gambling industry and its allies*. Routledge.

[CR49] Palmer, L., Cringle, N., & Clark, L. (2022). A scoping review of experimental manipulations examining the impact of monetary format on gambling behaviour. *International Gambling Studies*, 1–23. 10.1080/14459795.2022.2041067.

[CR50] Parke, J. (2009). *A medium to long-term programme of research for investigating gaming machines in Great Britain: Recommendations from international and British expert panels*.

[CR51] Parke, J., & Griffiths, M. D. (2007). The role of structural characteristics in gambling. In G. Smith, D. Hodgins, & R. Williams (Eds.), *Research and Measurement Issues in Gambling Studies* (pp. 211–243). Elsevier.

[CR52] Parke J, Parke A (2013). Does size really matter? A review of the role of stake and prize levels in relation to gambling-related harm. The Journal of Gambling Business and Economics.

[CR53] Pelham BW, Sumarta TT, Myaskovsky L (1994). The easy path from many to much: The Numerosity Heuristic. Cognitive Psychology.

[CR54] Rachlin H, Safin V, Arfer KB, Yen M (2015). The attraction of gambling. Journal of the Experimental Analysis of Behavior.

[CR55] Ramnerö J, Molander O, Lindner P, Carlbring P (2019). What can be learned about gambling from a learning perspective? A narrative review. Nordic Psychology.

[CR56] Rockloff M, Dyer V (2007). An experiment on the Social Facilitation of Gambling Behavior. *Journal of Gambling Studies / co-sponsored by the National Council on Problem Gambling and Institute for the study of*. Gambling and Commercial Gaming.

[CR57] Scharnowski, M., Scharnowskia, S., & Zimmermanna, L. (2021). *Fan tokens: Sports and speculation on the blockchain*. https://doi.org/http://dx.doi.org10.2139/ssrn.3992430

[CR58] Scholten, O. J., Hughes, N. G. J., Deterding, S., Drachen, A., Walker, J. A., & Zendle, D. (2019). Ethereum Crypto-Games: Mechanics, Prevalence, and Gambling Similarities. *Proceedings of the Annual Symposium on Computer-Human Interaction in Play*, 379–389. 10.1145/3311350.3347178

[CR59] Segal, D. (2022). *The Crypto Ponzi Scheme Avenger*. The New York Times. https://www.nytimes.com/2022/11/11/business/crypto-ponzi-scheme-hyperfund.html

[CR60] Shaffer HJ, Ladouceur R (2021). Moving away from individual responsibility: A comment. Journal of Gambling Studies.

[CR61] Shaffer HJ, LaPlante D, LaBrie R, Kidman RC, Donato AN, Stanton MV (2004). Toward a syndrome model of addiction: Multiple expressions, common etiology. Harvard Review of Psychiatry.

[CR62] Sinclair ESLL, Clark L (2021). Netflix’s ‘Gambling, explained’ and the Evolving Public Perception of Gambling. Critical Gambling Studies CGS Blog.

[CR63] Skinner, B. F. (1971). *Beyond freedom and dignity*. Knopf/Random House.

[CR67] Yani-de-Soriano M, Javed U, Yousafzai S (2012). Can an industry be socially responsible if its products harm consumers? The case of online gambling. Journal of Business Ethics.

[CR68] Zaucha T, Agur C (2022). Newly minted: Non-fungible tokens and the commodification of fandom. New Media & Society.

[CR69] Zendle D, Cairns P (2018). Video game loot boxes are linked to problem gambling: Results of a large-scale survey. PloS One.

[CR70] Zuber R, Yiu P, Lamb R, Gandar J (2005). Investor-Fans? An examination of the performance of publicly traded English Premier League Teams. Applied Financial Economics.

[CR64] Socios (2021). *The Fan Token Burn – Super Weekend*. Socios.Com. https://www.socios.com/the-fan-token-burn-super-weekend/

[CR65] Socios (2022a). *Socios.com to launch mobile games for fan token holders thanks to Wave partnership*. Socios.Com. https://www.socios.com/socios-com-to-launch-mobile-games-for-fan-token-holders-thanks-to-wave-partnership/

[CR66] Socios (2022b). *Socios - Fan tokens*.

